# Eye Movement Desensitization and Reprocessing for Adolescent Depression

**DOI:** 10.4306/pi.2008.5.1.60

**Published:** 2008-03-31

**Authors:** Hwallip Bae, Daeho Kim, Yong Chon Park

**Affiliations:** Department of Neuropsychiatry, Hanyang University Guri Hospital, Guri, Korea.

**Keywords:** Adolescent, Depression, Major depressive disorder, Eye movement desensitization and reprocessing, Psychotherapy

## Abstract

While cognitive behavior therapy is considered to be the first-line therapy for adolescent depression, there are limited data on whether other psychotherapeutic techniques are also effective in treating adolescents with depression. This report suggests the potential application of eye movement desensitization and reprocessing (EMDR) for treatment of depressive disorder related, not to trauma, but to stressful life events. At present, EMDR has only been empirically validated for only trauma-related disorders such as posttraumatic stress disorder. Two teenagers with major depressive disorder (MDD) underwent three and seven sessions of EMDR aimed at memories of stressful life events. After treatment, their depressive symptoms decreased to the level of full remission, and the therapeutic gains were maintained after two and three months of follow up. The effectiveness of EMDR for depression is explained by the model of adaptive information processing. Given the powerful effects observed within a brief period of time, the authors suggest that further investigation of EMDR for depressive disorders is warranted.

## Introduction

Depression in adolescents is associated with increases in the risk of suicide and substance abuse, poor psychosocial and academic functioning, and frequent comorbidity with other psychiatric disorders.^1^ Thus, early active and intense therapeutic interventions are needed for adolescents with depression. In the United States, the life time prevalence of major depressive disorder (MDD) in adolescents is expected to be 14%.^2^ Although Korean studies have used the self-report instruments to assess depression in adolescents, the results seem even more serious: one study of a national probability sample of adolescents reported 14% as the point prevalence between the ages of 15 and 19.^3^ Another study reported a prevalence of about 20% among middle and high school students.^4^

Despite the high prevalence and need for intensive treatment, there has been some debates surrounding the treatment of adolescent depression.^5^,^6^ The Food and Drug Administration (FDA) has recently warned physicians against the use of selective serotonin reuptake inhibitors (SSRIs), which are the mostly commonly prescribed antidepressants in adolescents or children due their association with an increased frequency of suicidal thoughts or behaviors.^5^

In the field of psychotherapy, there is also some debate over which treatment should be offered first. Controlled studies of adolescent depression are largely limited to cognitive behavior therapy (CBT); however, there has also been some evidence for the effectiveness of interpersonal psychotherapy (IPT) and family therapy.^7^ Earlier CBT studies reported its superiority over no treatment or waitlist controls. However, recent meta-analysis by Klein et al.^8^ revealed that the overall effect size of CBT is small in comparison studies with other effective psychotherapies, studies with more stringent methodology, and intent-to-treat analyses. Moreover, a clinical trial of fluoxetine, CBT, placebo, and combined fluoxetine-CBT in 439 adolescents showed that the effects of CBT alone were not different from those of placebo.^9^

At present, CBT is generally recommended as the first-line of treatment for adolescent depression and secondary pharmacotherapy is recommended for those not responding to CBT.^10^ However, studies on the application of other psychotherapies are scarce. Two controlled studies of IPT have been conducted: Mufson et al.^11^ reported that IPT was superior over monthly clinical monitoring, while Rosselló et al.^12^ found that both IPT and CBT were effective at follow-up. These studies suggest that psychotherapies other than CBT can be applied to adolescent depression. A recently developed psychotherapeutic technique, eye movement desensitization and reprocessing (EMDR) has received both clinical and academic attention for its effectiveness and rapidity in the treatment of posttraumatic stress disorder (PTSD).^13^ Although there is empirical evidence for the effectiveness of EMDR in PTSD and trauma-related disorders, studies on the use of EMDR for the treatment of other clinical disorders are scarce. As for depression, only anecdotal reports exist for children and adults.

To our knowledge, this study is the first report of the use of EMDR for adolescent depression, using formal diagnostic criteria and objective evaluation of symptoms. As depression is closely associated with negative life events, we hypothesized that processing the stressful memories preceding the onset of depression may reduce symptoms.

EMDR utilizes bilateral stimuli, such as horizontal eye movements, alternative tapping, or alternative sounds, to stimulate the information processing system of the brain in addition to effective methods of many established psychotherapies. It consists of a structured 8 phase protocol and is usually delivered in 90-minute weekly sessions.^14^

A typical session begins when therapists evaluate the image, cognition, emotion, and body sensations related to the traumatic memory. The patient and therapist also decide upon a positive cognition that may substitute for the negative and then check the scores of Subjective Units of Disturbance Scale (SUDS) and Validity of Cognition Scale. After this, patients are instructed to attend to the traumatic event and simultaneously follow the therapist's fingers (horizontal eye movements). This process of attending to both internal and external stimuli is called dual attention. If new materials come up after 28-30 eye movements, patients are once again asked to attend to the event and move their eyes. This dual attention and association are repeated until the early event is becomes no longer distressful.^14^

We report two cases of adolescent depression treated successfully with EMDR in a brief period. These patients had no history of trauma (even though the first case did involve the loss of the patient's father, it did not meet the Diagnostic and Statistical Manual of Mental Disorders-fourth edition (DSM-IV) criteria for a traumatic event^15^), thus this report suggests the potential use of EMDR for the treatment of non-trauma related disorders.

## Case

### Case 1

A 16-year-old high school girl visited the hospital in with the symptoms of depression, worry over her future, a difficulty with concentrating in class, and loss of will and appetite. Her father had died of the liver disease one year earlier; however, his death was expected, and she recalled that she was not shocked and felt rather neutral when it happened. At that time she carried on with life as normal and showed even improved academic performance.

Six months ago, she entered a boarding school. Around this time, she began to feel fatigued everyday activities became difficult for her with no explanation. She did not speak to her classmates, lost interests in school, and could not find the motivation to continue studying. At first, she thought this was due to the change in environment and the stress from her studies. Consequently, she moved to a new school so that she could continue living at home. However, this did not help, and her grades in school became worse. One month ago, she began to think of her father a lot, which often brought her to tears.

On her visit to our psychiatric outpatient unit, the second author (psychiatrist) evaluated her symptoms according to DSM-IV criteria and diagnosed her with MDD. At the same time, the same author administered the Hamilton Depression Rating Scale (HDRS)^16^,^17^ and she scored 18, which was indicative of moderate level of depression. In addition, to rule out the possible diagnosis of PTSD from her father's death, the Clinician-Administered PTSD Scale (CAPS)^18^,^19^ was administered. She did not fulfill the A2 criterion, as she did not react with fear, helplessness or terror. Symptom evaluation also did not satisfy the DSM-IV criteria for PTSD, and her total score on the CAPS was 26, which was under the diagnosable level.

As she and her mother did not want the pharmacotherapy and the therapist thought that the loss of her father contributing to the onset of depression could be a target for memory processing, the second author gave her the three sessions of EMDR therapy. The experimental nature of the use of EMDR treatment for depression was fully explained to the patient and her mother along with other treatment options before EMDR treatment was administered. Both the patient and her mother gave informed consent to the treatment.

In the first session, education about the EMDR process and history taking were carried out along with a 'safe place exercise', a guided imagery for the self-control of possible anxiety or distress between sessions. A positive resource was generated using her future as a successful career woman and this image was reinforced with sets of eye movement.

Her second session directly dealt with the memory of her father's death, and it began with the memory of viewing his dead body at the funeral and went through past everyday memories of her father with total of 25 sets. The association and process seemed smooth but toward the end, another distressing memory of being wounded at her elementary school came up, and the session was terminated incompletely. Her SUDS (0-10) score decreased from 6 to 4. She responded by saying that it is still distressing because 'it is a sad thing'. Though it may seem ecologically valid, the second session was terminated as incomplete.

**FIGURE 1:**
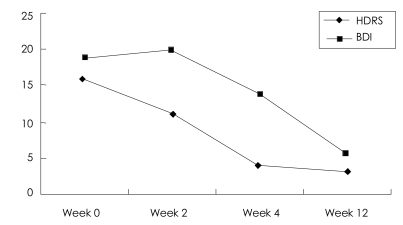
The scores of depressive symptoms at different times in case 1. Three sessions of EMDR were provided between week 0 and week 4. Follow-up examination was performed at week 12. HDRS: Hamilton Depression Rating Scale, BDI: Beck Depression Inventory, EMDR: eye movement desensitization and reprocessing.

In the third session, the memory of her father's death was reevaluated at the start of the session, and it still elicited a score of 2 or 3 on the SUDS. This became the target memory once again. The association went from the funeral to past happy times they had had as a family and to positive memories of her father. However, at the end of the session she complained of headache, and the session was terminated at her will. A total of 16 sets of eye movements had been conducted. As mentioned above, the memory processing was incomplete at the second and third session. But, later on, she reported that she thought of her father less frequently and that even when she did, she did not feel as sad as she had been feeling. At one week after the third session, she scored 4 on the HDRS and 6 on the Beck Depression Inventory (BDI)^20^,^21^ which were indicative of complete remission. Eight weeks later, she was followed up, and the remission was maintained (HDRS 3, BDI 6)(Figure 1).

### Case 2

A 14-year-old middle school student was referred to our out-patient department by a psychiatrist in private practice. She was tall and looked older than normal for her age. According to her mother, she had been a fine, intelligent, and optimistic child, who loved to spend time with her friends, and was always leading them and making jokes. Everyone liked her. However, two years earlier, father quit his stable job and started a small business. This sent her family into a financial crisis, and to make the matters worse, her father had an extramarital affair, quit his job and ran away with his mistress one year ago. The rest of the family had to move into a small apartment in a satellite city, and she also had to move to a new school.

Six months, ago when she met with her dad, she fainted (conversion?) but he told her not to pretend. Around this time of incident, she wrote a suicide note but did not attempt suicide. The last time she was his father was 5 months ago, and they had a quarrel and her father left saying "good luck" in a cynical tone. Around these two incidents, she had difficulty being around her friends and doing the academic work. She just could not bring herself to it, but she did not know why. She also showed weight loss (10 kg/3 months) with amenorrhea, decreased speech, interpersonal withdrawal, and a passive and avoidant attitude. Her symptoms began to worsen. She and her mother went to see a psychiatrist who thought that she needed some form of psychotherapy rather than pharmacotherapy alone and referred her to our clinic.

She did not have a history of major trauma in her life. She had good premorbid adjustment, as demonstrated by superior academic achievement and stable interpersonal relationships. She had a suicidal thought but made no plans or attempts at suicide. She had active support from her sister and her mother. Although she showed the biological symptoms of depression, her depression seemed to have a strong experiential component arising after a stressful life event, which was the separation from father. Finally, he and her mother did not want to be treated with medication but preferred psychotherapy. On these accounts, EMDR was administered by the second author. The experimental nature of the use of EMDR for depression was explained to the patient and her mother, along other treatment options, and both the patient and her mother gave informed consent to the treatment.

She was diagnosed with MDD according to DSM-IV criteria and scored 26 on the HDRS administered by the therapist, which was indicative of severe depression. She then completed seven sessions of EMDR.

In the first session, she presented her three worst memories, which were all related to the recent stressful events including: 1) having to move and be separated from her friends; 2) her father's extramarital affair; 3) her father's decision to start a new career, which started the whole situation. As a safe place, she was reminded of her childhood house and this was maintained positively, with a slight sense of anxiety. The first session targeted the memory of having to leave her old friends, and she processed it smoothly and finished with the emotion of happiness. However, her SUDS score remained unchanged.

Before the start of the second session, her mother told the therapist that she had stopped weeping after the first session. The second and third sessions were aimed at her father's extramarital affair, and the therapist intervened to express anger toward her father. The sessions proceeded well, but once again her SUDS score did not improve. This led the therapist to believe that the subjective evaluation of distress was not reliable or valid to this patient.

Before the fourth session, the scales were reevaluated and the results showed that her scores were lowered by more than 50% of their initial value (HDRS 26 to 11, BDI 42 to 21). Her symptoms improved to the level of mild depression. The patient reported that she felt better, talked to her classmates and had less fear in relationships. She added that people were wondering why she was improving. The fourth session targeted recent memories of her relationships with peer, which improved the estrangement from her friends as a result of the depression. When desensitizing, she associated changes in her symptoms retrospectively and chronologically and was finally able to accept that it was her symptoms that were causing her difficulty with interpersonal relationships and not her stupidity, as she had previously thought.

After the fourth session, she had an emotional outburst after confronting new people at her new church, and this incident became the target of the fifth session. This was a different picture of externalization after she became depressed. After desensitization, the fear of new people decreased and she began to experience a sense of hope. In the sixth session, the therapist and patients probed for any negative feeder memory, and she recalled an incident at the age of six, when her mother broke her doll in a fit of anger. This memory was not processed completely.

**FIGURE 2:**
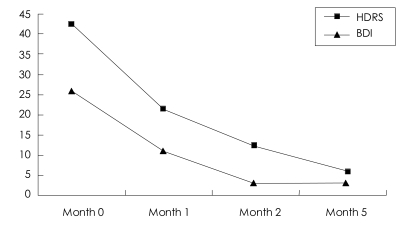
The scores of depressive symptoms at different times in case 2. Seven sessions of EMDR were provided between month 0 and 2. Follow-up examination was performed at month 5. HDRS: Hamilton Depression Rating Scale, BDI: Beck Depression Inventory, EMDR: eye movement desensitization and reprocessing.

The seventh and final session processed her academic success as a future template. After the completion of treatment, she confessed that there was nothing she could do change the fact that her father had an affair and that she would leave it to God. One week later, after the seventh session, she scored 3 on the HDRS, which was indicative of complete remission of MDD, and 12 on the BDI. At the 3-month follow-up, her HDRS score was 3 and her BDI score further decreased to 6 (Figure 2).

## Discussion

Depression is a multifactorial illness, and it was suggested that stressful life events may cause the condition.^22^ Research also indicates that loss and humiliation (especially separation initiated by others), as experienced by our patients are most depressogenic stressful life events.^23^ In our study, one patient began to suffer from depression six months after her father's death, while the other patient began to suffer from depression six months after her father had an extramarital affair. Processing memories related to negative experiences brought about symptomatic improvement, suggesting the causative role of stressful events in the development of depression.

Adjustment disorder with depressive symptoms was the first condition to be considered in the different diagnoses of our patients. However, adjustment disorder begins at most within three months after the stressful events, which does not correspond with the onset of depression in these two cases. Moreover, they met the full criteria for MDD and were severe enough to be categorized as moderate and severe depression, respectively. The possibility of bereavement needed to be considered in the first case, but the condition had developed at six months after her father's death.

To our knowledge, there are four anecdotal reports on depression, which included cases of: 1) an adult with depression who underwent EMDR for childhood trauma during the course of psychoanalytic psychotherapy^24^; 2) postpartum depression^25^; 3) senile depression.^26^; 4) child depression.^27^ These reports did not specify any use of diagnostic criteria in the diagnosis of depression. In addition, psychological instruments were not used in any of these cases, except for the case of senile depression and concurrent treatments (medication and other psychotherapy) were given in addition to EMDR in all cases, except the case of childhood depression.

Despite the difficulty of generalizing these findings, these cases all suggest that the targeting of the early childhood memories, which were the causes of the current symptoms. consequently reduced the current level of depression. For instance, Parnell^25^ described a woman with postpartum depression who underwent 12 sessions of EMDR, which resulted in the complete remission of her symptoms of anxiety and depression. In this case, the patient's symptoms improved after processing an early childhood memory of having to take care of her baby sister and wishing to kill her.

In contrast, our cases dealt only recent memories, except for case 2, in which a childhood memory of her mother was targeted in the sixth session. However, it is uncertain that this further contributed to her improvement because she had already shown much progress at the reevaluation. Further studies are needed to investigate whether or not focusing on only recent life events alone will have adequate therapeutic effects.

The relative briefness of treatment in our patients needs to be addressed. In these cases, mild to moderate depression required only three sessions of EMDR and moderate to severe depression required seven sessions. At this time, it is not possible to generalize this finding. However, it is noteworthy that established psychotherapies for depression, such as CBT or ITP require a mean of 12 sessions plus extra homework. The rapid treatment effect observed with EMDR in comparison to exposure-based therapy was noted in a previous study of PTSD,^28^ however, the reason behind this finding and why EMDR works in depression need further discussion.

Shapiro^29^ explained the effect of EMDR as 'adaptive information processing': the brain has its own physiological information processing system, which generally directs any new information to a more adaptive state. But when the information from distressing or traumatic experiences is not sufficiently processed enough, it remains as a distorted thought or perception in its originally input form. This distressful memory causes a concurrent dysfunctional reaction, and the brain can adaptively process it with eye movements or other bilateral stimuli.

In this context, stressful but non-traumatic memories can also be processed to a more adaptive and positive state with EMDR. Through EMDR, the first patient was able to process the memory of her father's death linked with sadness and helplessness, and the second patients was able to process the memory of her father's extramarital affair linked with anger and humiliation with more adaptive, objective, distancing, and positive associations.

It is particularly interesting to note that the stressful life events targeted in this report were all related to interpersonal relationships. More specifically, the second case dealt with relational aspects: old friends, current peers, parents, and new people. This type of approach is similar to what IPT postulate about depression and their approach to treating depression. However, a case series with a longer follow-up and controlled studies are needed to demonstrate the effectiveness of EMDR for depression. Further study is also needed to confirm which targets work best in the treatment of depression with EMDR and how many sessions are actually needed.

The limitations of this study include its nature as a case report, the lack of a third party evaluator, and its short follow-up period. The fat that the HDRS and BDI have not been validated for use in adolescent populations should also be considered when interpreting the findings of this study. However, even in research, many adult instruments have been used to assess depression in adolescents.^30^ The findings of this study should be carefully accepted, as these patients were not severe enough to be hospitalized or treated with medication. However, the efficiency (fewer sessions and no home work) and effectiveness of treatment in these cases need further generalization.
